# Comparative Studies of Different Preservation Methods and Relative Freeze-Drying Formulations for Extracellular Vesicle Pharmaceutical Applications

**DOI:** 10.1021/acsbiomaterials.3c00678

**Published:** 2023-09-06

**Authors:** Francesca Susa, Tania Limongi, Francesca Borgione, Silvia Peiretti, Marta Vallino, Valentina Cauda, Roberto Pisano

**Affiliations:** † Department of Applied Science and Technology (DISAT), Politecnico di Torino, Corso Duca degli Abruzzi 24, 10129 Turin, Italy; ‡ Consiglio Nazionale delle Ricerche di Torino, Strada delle Cacce 73, 10129 Turin, Italy

**Keywords:** extracellular vesicles, excipients, freeze-drying, storage, human serum, cell culture media, secretome, active pharmaceutical ingredient

## Abstract

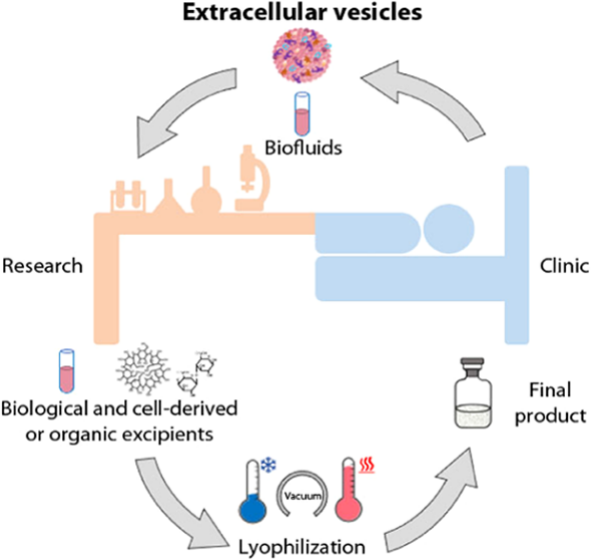

Extracellular vesicles (EVs) have been studied for years for their role as effectors and mediators of cell-to-cell communication and their potential application to develop new and increasingly performing nanotechnological systems for the diagnosis and/or treatment of many diseases. Given all the EVs applications as just isolated, functionalized, or even engineered cellular-derived pharmaceuticals, the standardization of reliable and reproducible methods for their preservation is urgently needed. In this study, we isolated EVs from a healthy blood cell line, B lymphocytes, and compared the effectiveness of different storage methods and relative freeze-drying formulations to preserve some of the most important EVs’ key features, i.e., concentration, mean size, protein content, and surface antigen’s expression. To develop a preservation method that minimally affects the EVs’ integrity and functionality, we applied the freeze-drying process in combination with different excipients. Since EVs are isolated not only from body fluids but also from culture media conditioned by the cells growing there, we decided to test both the effects of the traditional pharmaceutical excipient and of biological media to develop EVs solidified products with desirable appearance and performance properties. Results showed that some of the tested excipients, i.e., sugars in combination with dextran and glycine, successfully maintained the stability and integrity of EVs upon lyophilization. In addition, to evaluate the preservation of the EVs’ biological activity, we assessed the cytotoxicity and internalization ability of the reconstituted EVs in healthy (B lymphocytes) and tumoral (Burkitt’s lymphoma) cells. Reconstituted EVs demonstrated toxicity only toward the cancerous cells, opening new therapeutic opportunities for the oncological field. Furthermore, our study showed how some biological or cellular-conditioned fluids, commonly used in the field of cell cultures, can act not only as cryoprotectants but also as active pharmaceutical ingredients, significantly tuning the therapeutic effect of EVs, even increasing their cellular internalization.

## 1 Introduction

Extracellular vesicles (EVs) have increasingly been recognized as crucial intercellular communication mediators.^1^ Thanks to their important role in cellular physiology and pathology, they have recently been also proposed as promising biomarkers for early disease detection and as innovative therapeutic agents, e.g., as drug or gene delivery vehicles.^2−6^ However, despite the increasing use of EVs in basic, clinical, and translational research, advanced knowledge and optimization are still needed in their storage methods and formulations.^7−9^ The preservation of EVs aims to simplify their transportation and handling, while keeping their intrinsic physical and biochemical characteristics unvaried. At present, indeed, the only effective storage method that maintains EVs’ features is freezing at −80 °C, which introduces high costs and limits transportability and availability for large-scale clinical trials and therapeutic applications.^10^

EVs are constituted by a phospholipid bilayer containing specific lipids and proteins for signaling, targeting, and other specific functions, which display a high temperature-dependent fluidity.^11^ This bilayer can be strongly affected by the physical and chemical stresses of the external environment such as the formation of ice crystals, pressure variations, and drying stresses. Furthermore, EVs have an inner core containing an aqueous solution rich in proteins, RNAs, and other cytosolic compounds. This solution can also form crystals upon freezing and be susceptible to osmotic changes, affecting their integrity, i.e., the original morphological structure of EVs.^12^

Many researchers have already investigated the impact of storage conditions on the intrinsic characteristics of EVs in terms of stability, particle number, diameter, and biological function.^13,14^ One of the most used methods to preserve EVs is cryopreservation, even if it is usually associated with the so-called cryoinjury due to the osmotic imbalance and intracellular ice formation. During freezing, the growth of ice crystals causes the exclusion and thus the dramatic increase of the concentration of excipients and EVs. Hence, EVs are progressively transported away from the freezing front by diffusion remaining confined within highly concentrated unfrozen medium, resulting in an osmotic gradient, promoting the water flow out of the EVs through exosmosis. This phenomenon is known as cryo-concentration and may cause changes in ionic strength, osmolarity, and pH.^15^

The impact of storage conditions on the morphofunctional integrity of EVs is strongly related to their tissue or cells of origin. For instance, the degradation of EVs in a urine sample takes place after 2 h from the collection,^16^ but they are well preserved at −80 °C.^17^ EVs from blood seem to be more resistant to long-term storage and freeze–thaw; storage of plasma at +4, −20, and −80 °C or room temperature (RT) for a few days does not result in significant degradation of EVs,^18,19^ while Akers et al. proved the stability of cerebrospinal fluid-derived EVs considering preservation at both RT and −80 °C for a few days.^20^ EVs from cardiosphere-derived cells are stable after 1 week of storage at +4, −20, and −80 °C. However, the microRNA (miRNA) levels carried by EVs decrease during this period if EVs are stored at +4 or −20 °C and continue to decrease with the storage time. In contrast, at −80 °C, the miRNA levels undergo little changes.^21,22^ For human neutrophilic granulocyte-derived EVs, the best storage temperature is −80 °C to prevent significant changes in physical and functional properties compared to RT, +4 and −20 °C, compared to the same period of time of 1 month.^23^

To overcome damages associated with freezing, cryoprotectants can be added to EVs suspensions, being characterized by high water solubility and low toxicity, e.g., dimethyl sulfoxide^24^ and trehalose.^14,15,25^

More recently, lyophilization has been proposed as an alternative method of cryopreservation for EVs storage.^26^ This three-step preservation method is widely used in the pharmaceutical industry to preserve thermolabile materials such as vaccines, viruses, proteins, peptides, and colloidal carriers. In addition, freeze-dried materials can often be stored at RT and quickly reconstituted in water or physiological solution.^15,27^ Nevertheless, stresses occurring during the lyophilization process, i.e., ice crystal formation, cryoconcentration, dehydration, swelling of the amphiphilic molecules, and osmotic effects, may damage the EVs’ structure, cargoes, and membrane’s composition.^20,22,28,29^

Various authors investigated the effect of the freeze-drying process on EVs from various sources, and as previously described for cryopreservation, they observed that the stability of lyophilized EVs depends on their origin.^28−30^ Besides that different researchers freeze-dried EVs without additives,^29,31,32^ in general, to successfully maintain the original vesicles’ features, lyophilization of EVs from different sources requires the addition of cryo- and lyoprotectants to preserve them during the process. One of the most widely used protecting agents for freeze-drying is trehalose; lyophilized EVs with trehalose, alone or in combination with other compounds, such as poly(vinylpyrrolidone) 40,^33^ were similar to the ones stored at −80 °C in terms of size, morphology, concentration, and content of proteins and RNAs.^30,34,35^ The addition of sucrose to the EVs formulation before freeze-drying allowed the maintenance of their original features after the rehydration.^26,36^ Mannitol showed fewer promising results than trehalose and sucrose for the maintenance of the original EVs’ concentration and morphology upon lyophilization.^30,37^ In contrast, poly(ethylene glycol) (PEG) did not demonstrate good stabilization behavior for EVs, since it induced the aggregation of particles, probably by crosslinking the vesicles.^30^

In this work, the effects of different temperatures on short- and long-term storage on B lymphocyte-derived EVs were investigated for the first time considering vesicles’ concentration, protein content, and membrane immunophenotyping. With the aim to overcome the limits introduced by cryopreservation and to open new perspectives for the bench-to-bed translation of EVs-based therapeutics, the freeze-drying process was here applied to EVs. This is the first time that lyophilization was applied to B lymphocyte-derived EVs, evaluating not only the morphological variations induced by the process but also the biological effects in an in vitro model. B lymphocyte-derived EVs were lyophilized with or without the addition of already commonly used excipients, such as sucrose and trehalose. In addition to these conventional lyoprotectants, we tested, in an innovative way, the effectiveness of some biological or cellular-conditioned fluids to ensure the morphofunctional integrity of EVs during the freeze-drying process. Since many of the EVs used as biomaterials for nanomedical and drug delivery applications are obtained by sterile differential ultracentrifugation from conditioned cell culture media, we decided to test the effect of different biological free-cell media as bioderived excipients to increase the possibilities of maintaining the biological activity unaltered during the various steps of the conservation processes.

Therefore, this work paves the way for the use of new biological excipients with the aim to develop increasingly biomimetic nanotechnological tools for pharmaceutical applications. The lyophilized products were characterized by electron microscopy, nanoparticle tracking analysis, and residual water content analysis and finally tested to assess any cytotoxicity and internalization potential using both healthy (B lymphocytes) and tumoral (Burkitt’s lymphoma) cell line models.

## 2 Experimental Section

### 2.1 Cell Cultures

Cell lines were cultured at 37 °C under a 5% CO_2_ atmosphere in 75 cm^2^ nontreated cell culture flasks (Corning, Corning, NY). Cell culture media were supplemented with heat-inactivated fetal bovine serum (FBS, obtained by heating at 56 °C for 30 min) and 1% of penicillin/streptomycin (P/S, 10 000 units penicillin and 10 mg streptomycin/mL, Sigma, Darmstadt, Germany).

Lymphocytes (IST-EBV-TW6B) were purchased from the cell bank IRCCS AOU San Martino IST (Genova, Italy). They were cultured in advanced Roswell Park Memorial Institute (RPMI) 1640 cell culture medium (Gibco, Thermo Fisher Scientific, Waltham, MA) complemented with 20% FBS (Gibco) and 1% l-glutamine 200 mM (Q, Lonza, Basel, Switzerland), maintaining the cell density between 9 × 10^4^ and 9 × 10^5^ cells/mL. After 20 days of use, the cell culture medium was supplemented with 1% Q and 1% of non-essential amino acid solution (Sigma).

The Daudi cell line (ATCC CCL-213TM), derived from the peripheral blood of a Burkitt’s lymphoma patient, was maintained in RPMI 1640 culture medium (ATCC), with a cell density between 3 × 10^5^ and 3 × 10^6^ cells/mL.

### 2.2 Extracellular Vesicles Isolation

Lymphocyte-derived EVs were isolated from 72 h-conditioned medium of lymphocytes cultured in advanced RPMI 1640 cell culture medium with 20% of EVs-free FBS, 1% Q, and 1% P/S. EVs were removed from serum by ultracentrifugation, FBS was ultracentrifuged at 100 000*g*, 4 °C overnight, with an Optima MAX-XP ultracentrifuge and an MLA-50 rotor (Beckman Coulter, Brea, CA), and the obtained supernatant was the depleted FBS (dFBS).

For the isolation of EVs, 1.5 × 10^5^ lymphocytes/mL were plated in 75 cm^2^ nontreated flasks in a total volume of 200 mL of culture medium containing dFBS and maintained in culture for 72 h.

The EVs isolation protocol was adapted from Théry et al.,^38^ based on sterile differential ultracentrifugation. To reduce the occurrence of apoptotic bodies, before the isolation, the cells’ viability was assessed and only samples with ≥90% viable cells were further processed.

In detail, after 3 days in vitro, the conditioned culture medium was collected and centrifuged at 150*g* for 10 min to pelletize viable lymphocytes from the solution. Next, the obtained supernatant was centrifuged at 2000*g* for 20 min to remove dead cells. Then, the supernatant was centrifuged at 10 000*g* for 30 min to discard the cells’ debris. The supernatant was collected, transferred in ultracentrifuge tubes (32 mL, OptiSeal tubes, Beckman Coulter), and ultracentrifuged at 100 000*g* for 70 min. The tubes’ resulting pellets were carefully recovered, resuspended in cold 0.1 μm-filtered phosphate-buffered saline (PBS) solution, and reunited in a single ultracentrifuge tube. This was ultracentrifuged at 100 000*g* for 60 min. The final pellet was recovered by resuspension in 600 μL of 0.1 μm filtered physiological saline solution (PS, 0.9% NaCl; NovaSelect, Tito Scalo (PZ), Italy).

### 2.3 Extracellular Vesicles Characterization

Lymphocyte-derived EVs were characterized using different techniques, as follows, to have a comprehensive overview of the main features of the vesicles.^8^

#### 2.3.1 Transmission Electron Microscopy

The morphology of EVs was investigated with transmission electron microscopy (TEM). For the analysis, a drop of 7 μL of the sample was deposited on carbon and Formvar-coated 400 mesh grids (Ted Pella, Redding, CA) previously glow-discharged with an Edwards E12E vacuum coating unit (Burgess Hill, U.K.). After 5 min of incubation to allow the adsorption of EVs, the grids were rinsed several times with water and negatively stained with aqueous 0.5% w/v uranyl acetate. The excess solution was then removed with filter paper.

Observations and photographs were obtained using a Philips CM 10 transmission electron microscope (Eindhoven, The Netherlands), operating at 60 kV. Micrograph films were developed and digitally acquired with a D800 Nikon camera at high resolution. Images were trimmed and adjusted for brightness and contrast, and the scale was set using ImageJ 1.53c version.

#### 2.3.2 Nanoparticle Tracking Analysis

Lymphocyte-derived EVs sample concentration and size distribution were evaluated through the nanoparticle tracking analysis (NTA) technique using a Nanosight NS300 (Malvern Panalytical, Malvern, U.K.) equipped with a laser beam with λ = 505 nm, a Nanosight syringe pump, and NTA 3.4 software.

Before the analysis, all the samples were diluted to a final volume of 500 μL in physiologic solution, reaching a final concentration between 1 and 5 × 10^8^ particles/mL to maintain the particles/frame index between 20 and 100, the optimal working range for the instrument. The measurements were carried out by acquiring three videos of 60 s each, with an infusion pump rate of 50 au, the screen gain at 1, and the camera level between 15 and 16. Software then analyzed the videos with the detection threshold set at 5.

#### 2.3.3 Protein Content Analysis

The protein content of the EVs samples was quantified with the colorimetric Bradford assay, based on the dye Coomassie brilliant blue G-250. First, the set of standards was prepared using bovine serum albumin (BSA, Sigma) diluted in PBS at different concentrations: 0, 5, 10, 15, 20, 25, 40, 80, 100, and 160 μg/mL. The analysis was carried out in a 96-well flat-bottom plastic culture plate (Greiner Bio-one, Kremsmünster, AT): in each well, for the calibration curve, 10 μL of each standard concentration and for the samples, 5 μL of PBS and 5 μL of the EVs sample were plated in triplicate. Then, the Coomassie brilliant blue (protein assay dye reagent concentrate, Bio-Rad, Hercules, CA) was diluted 1:5 in bidistilled water, and 200 μL was added to each well with the standard or sample drop. The absorbance at 590 nm was read on a spectrophotometer (Multiskan Go microplate spectrophotometer, Thermo Fisher Scientific). The calibration curve was plotted using a linear fitting, and the protein concentration was determined from the equation of this curve.

#### 2.3.4 Immunophenotypical Characterization

The expression of the surface antigens CD63 and CD81, typical exosomal markers, and CD20, a lymphocytes-distinctive marker,^39^ was evaluated using flow cytometry. For the analysis, 2.5 μg of EVs proteins, measured by Bradford assay, was incubated with 5 μL of aldehyde/sulfate latex beads, 4% w/v, 3 μm (Thermo Fisher) for 15 min at RT. Then, the solution was diluted to 500 μL with PBS and incubated for 2 h at RT on a tube rotator at 20 min^–1^ to allow the coupling of the EVs to the beads’ surface. To saturate any free binding site, 55 μL of PBS/1 M glycine was added to the solution and incubated for 30 min at RT. Next, the samples were centrifuged for 3 min at 4000 rpm, the supernatants were discarded, and the bead pellets were resuspended in 250 μL of PBS/0.5% BSA (w/v). Next, a proper number of beads were incubated with the allophycocyanin (APC) conjugated antibody CD81 (CD81-APC, Miltenyi Biotec, Bergisch Gladbach, DE) or the phycoerythrin (PE)-conjugated antibody CD20 or CD63 (CD20-PE, CD63-PE, Miltenyi Biotec, Bergisch Gladbach, DE) and the respective isotype controls for 30 min in the dark at 4 °C. Then, two washing steps were performed in 200 μL of PBS/BSA. Samples were investigated with a Guava Easycyte 6–2L flow cytometer (Merck Millipore, Burlington, MA). To adjust the instrument voltages and gate the beads’ population, excluding debris and impurity, unstained beads were used. As a result, 5000 gated events were acquired in a very low modality (0.12 μL/s flow rate). The APC signal was collected using the red laser (642 nm, Red-R Channel), while PE was collected with the blue laser (488 nm, Yellow- B channel). Results were analyzed with Guava Incyte Software (Merck Millipore) in terms of median fluorescence intensity (MFI) of the antigen minus the MFI of the isotype control^39,40^ and histograms plotted using FCS Express 6 software. The experiment was repeated five times (*n* = 5) and reported as mean ± standard error (SE).

### 2.4 Evaluation of the Storage Parameters and Their Effects on the Extracellular Vesicles’ Size and Biocomposition

To provide a thorough study on the EVs preservation, the influence of various combinations of temperature and time of storage was investigated and compared with the just isolated sample and the most widely used method to preserve EVs, i.e., freezing at −80 °C.

#### 2.4.1 Parameter Setting Validation

After the isolation of EVs, the effect of different storage temperatures and times was tested. In detail, the considered storage times were 1 day, 1 week, and 1 month, while the conditions evaluated are shown in Table 1. Different aliquots were prepared for each storage time, to avoid the freezing and thawing of EVs (for sub-zero temperatures), which would be detrimental for the maintenance of their structure and properties.^41^

**Table 1:** List of the Preservation Methods Tested at Different Time Points

preservation methods
quench freezing and storage at –80 °C
freezing in liquid nitrogen and then stored at –80 °C
shelf-ramped freezing at 1 °C/min down to –80 °C and then storage at –80 °C
quench freezing and storage at –20 °C
storage at 4 °C
storage at RT
storage at 37 °C

#### 2.4.2 Characterization of Extracellular Vesicles at Different Time Points

After 1 day, 1 week, or 1 month, an aliquot of EVs was analyzed through NTA, Bradford assay, and CD20 antigen’s expression to evaluate the effect of the storage method on the EVs’ stability, morphology, integrity, and composition.

Since different EVs isolations were considered, each experiment was calculated as a percentage of the fresh, just isolated sample to make comparisons consistent among each other. Independent experiments were carried out in triplicate.

### 2.5 Freeze-Drying of Extracellular Vesicles

#### 2.5.1 Preparation of the Formulations for EVs Freeze-Drying

During EVs lyophilization, various excipients were used, alone or in combination, as cryo- and lyoprotectants. Among others are lactose (Fluka, Charlotte, NC), sucrose (Fluka, Charlotte, NC), trehalose (Sigma), and h-β-cyclodextrin ((2-hydroxypropyl)-β-cyclodextrin, molecular weight = ∼1460 kDa, Aldrich Chemistry) as stabilizers, mannitol (Chem-Lab, Zedelgem, Belgium) and glycine (Sigma-Aldrich) as bulking agents, and dextran 40 (molecular weight = ∼40 000 g/mol, PanReac AppliChem) as a collapse temperature modifier. All excipients were added at a concentration of 5 and 10% (w/v) to the EVs suspensions in physiologic solutions. Furthermore, since dextran, PEG (molecular weight = ∼400 g/mol, Sigma-Aldrich), and glycine are often used in combination with polysaccharides, solutions with 7% of lactose/sucrose/trehalose and 3% of dextran 40, PEG, or glycine and with 7% of dextran and 3% of PEG were also investigated. All the listed excipients were added to EVs in physiologic solution. In addition, some formulations of biological origin were also tested to evaluate their suitability as cryo- and lyoprotectants. In detail, EVs-free human serum (Sigma) decomplemented or not, advanced RPMI 1640 cell culture medium, and the EVs-free conditioned culture media (secretome) after 3 days of culture with B lymphocytes were added to the EVs in PS at the concentration of 10 or 50% (w/v). EVs-free human serum and EVs-free secretome were obtained by overnight ultracentrifugation at 100 000*g* at 4 °C. All the formulations are shown in Table 2.

**Table 2:** List of the Formulations Used for the Freeze-Drying of EVs^a^

excipient	concentration (w/v) (%)	abbreviation
physiologic solution		PS
lactose	5	L5%
	10	L10%
sucrose	5	S5%
	10	S10%
trehalose	5	T5%
	10	T10%
H-β-cyclodextrin	5	C5%
	10	C10%
dextran	5	D5%
	10	D10%
lactose–dextran	7–3	L-D
sucrose–dextran	7–3	S-D
trehalose–dextran	7–3	T-D
dextran–PEG	7–3	D-PEG
lactose–PEG	7–3	L-PEG
sucrose–PEG	7–3	S-PEG
trehalose–PEG	7–3	T-PEG
mannitol	5	M5%
	10	M10%
glycine	5	G5%
	10	G10%
lactose–glycine	7–3	L-G
sucrose–glycine	7–3	S-G
trehalose–glycine	7–3	T-G
cells and EVs-free human serum	10	HS10%
	50	HS50%
decomplemented, cells and EVs-free human serum	10	HSDC10%
	50	HSDC50%
advanced RPMI 1640	10	RPMI10%
	50	RPMI50%
cells and EVs-free secretome (100k fraction of secretome)	10	Secretome10%
	50	Secretome50%

a All the excipients were dispersed in physiologic solution.

#### 2.5.2 Thermal Characterization of the Lyoformulations

All the solutions were characterized by differential scanning calorimetry (DSC) and freeze-drying microscopy (FDM) to determine the maximum allowable temperature beyond which they underwent undesired phenomena during primary drying. DSC analysis was performed to determine the glass transition temperature of the frozen sample (*T*_g_′) and the eutectic melting temperature (*T*_eu_), while FDM analysis was performed to estimate the collapse temperature (*T*_c_).^42^

For these analyses, only solutions with excipients alone were tested since the EVs concentration was very low and their contribution to the thermal behavior of the frozen samples was negligible.

All the formulations were first thermally characterized by differential scanning calorimetry (DSC Q200, TA Instruments, New Castle, DE). A stainless-steel pan (Tzero, TA Instruments, New Castle, DE) was filled with ∼20 μL of the solution, sealed, and compared with an empty pan as reference. Samples were frozen by cooling them to −60 °C at 1 °C/min and heating them up to 20 °C at 5 °C/min.

The FDM (Type BX51, Olympus Europe, Hamburg, Germany), equipped with a PE95-T95 temperature controller (Linkam, Scientific Instruments, Tadworth, Surrey, U.K.) and a vacuum pump, was used to detect the collapse temperature of all the formulations.

#### 2.5.3 Freeze-Drying Protocol

The freeze-drying process was divided into three phases: freezing, primary drying, and secondary drying. First, the samples were frozen at −80 °C in an ultralow-temperature freezer and then dried using a REVO pilot-scale freeze dryer (Revo series, Millrock Technology, Kingston, NY). Chamber pressure was detected through a capacitive sensor (Baratron, 626A model, MKS Instruments, Andover, MA) and a thermal conductivity gauge (Pirani, PSG-101-S model, Inficon, Bad Ragaz, Switzerland). The Pirani sensor was sensitive to the gas composition, and despite maintaining constant total pressure, the detected signal can vary during primary drying since the gaseous composition varied from almost 100% of water during sublimation to 100% of inert or air atmosphere when the ice sublimation was concluded, and the water vapor generation stopped. Thus, when the ratio of Pirani to Baratron reached unity, it was determined as the end of primary drying.^43^

The freeze dryer shelves were precooled at −50 °C; then, samples prefrozen at −80 °C were loaded inside the chamber, and primary drying was launched. At the end of freezing, the shelf temperature was progressively increased at 0.5 °C/min and the primary drying was carried out at 100 μbar and −10 °C. At the end of primary drying, determined as the time at which the Pirani-to-Baratron pressure ratio was 1, the shelf temperature was increased to 20 °C at 0.5 °C/min and held for 12 h. Then, the vacuum was released at the atmosphere value by nitrogen injection inside the freeze dryer chamber.

#### 2.5.4 Characterization of the Freeze-Dried Extracellular Vesicles

Before their reconstitution, the lyophilized EVs were characterized in terms of cake appearance and residual moisture (RM).

The cake’s appearance is the most subjectable critical quality attribute of lyophilized products and is evaluated by visual inspection. Ideally, the cake should preserve the same shape and size as the frozen product, with uniform color and texture. Despite such expectations, there are no systematically defined criteria to accept or reject a cake with variations that deviate from the misunderstood term “elegant”. Acceptable and unacceptable cake appearances are determined based on historical precedent. Many terminologies were coined to describe variations in the cake appearance deviating from the definition of “uniform and elegant”, i.e., collapsed cakes, that resulted in a reduced specific surface area, melted-back cakes, shrinkage, etc.^44^

The residual moisture of the lyophilized samples was investigated by the Karl Fischer titration (Karl Fischer Moisture Meter CA-31, Mitsubishi, Japan). The hydranal titration solvent (Sigma-Aldrich, Milano, Italy) was used to reconstitute most of the formulations to be analyzed. In contrast, formamide was added to those samples containing dextran, glycine, and mannitol, as they are scarcely soluble in methanol. A titration blank was carried out in triplicate to determine the moisture concentration in the water standard and formamide.

#### 2.5.5 Characterization and Biological Behavior of the Reconstituted Freeze-Dried Extracellular Vesicles

Before the biological assays, freeze-dried EVs samples were reconstituted in serum-free lymphocyte culture medium. For optimal reconstitution, after adding the media, the samples were left for 30 min at 4 °C. After that, the samples were divided into two aliquots, one for uptake and one for cytotoxicity and NTA analyses. The aliquot used for the uptake was labeled by adding 1 μL of wheat germ agglutinin Alexa Fluor 647 conjugate (WGA647, Thermo Fisher, λ_ex_ = 650 nm, concentration of the stock solution 0.1 mg/mL in PBS) to each 100 μL of EVs. Both aliquots were incubated for 30 min at 37 °C with 180 rpm shaking. Then, the samples were ultrafiltered with Amicon Ultra 50 kDa centrifugal filters (Merck Millipore) to concentrate EVs and remove the unbound dye.

For the TEM analyses, the unlabeled EVs of the sample with only PS were resuspended in 0.1 μm filtered physiologic solution and imaged as described above.

For the NTA analyses, a proper amount of unlabeled EVs was resuspended in a total volume of 45 μL of 0.1 μm filtered physiologic solution and before the analysis further diluted at 1:100 or 1:200. The analyses were repeated at least twice and normalized to the results obtained for the EVs stored at −80 °C as mean ± SE.

For the cytotoxicity assays, a proper amount of unlabeled EVs was collected from the Amicon-eluted solution and resuspended in cell culture medium to reach the concentration of 10 μg/mL. 2 × 10^5^ lymphocytes and Daudi for each mL of treatment solution were centrifuged and resuspended in the EVs solution and 100 μL was plated in each well of a 96-well flat-bottom plastic culture plate. After 20 h of incubation at 37 °C and 5% CO_2_, 10 μL of WST-1 reagent (CELLPRO-RO, Roche, Basel, CH) was added to each well, and after further 4 h of incubation, the formazan absorbance was detected at 450 nm through a microplate spectrophotometer (Multiskan Go microplate spectrophotometer, ThermoFisher Scientific, Waltham, MA) using a 620 nm reference. Independent experiments were carried out in duplicate and at least twice for each cell line, and the results were normalized to the untreated sample.

For the uptake, eluted WGA647-labeled EVs were resuspended in the correspondent cell culture media to obtain a final protein concentration of 10 μg/mL. The assay was carried out in the same way as cytotoxicity, where 2 × 10^5^ cells for each mL of the treated solution were centrifuged and resuspended in the treatment solutions and 250 μL was plated in a well of a nontreated 96-well plate and incubated for 24 h at 37 °C under a 5% CO_2_ atmosphere. After incubation, cells from the different wells were collected and washed twice in PBS by centrifuging and resuspending them in 350 μL of PBS.

Flow cytometric analyses were performed with a Guava Easycyte 6–2L flow cytometer (Merck Millipore), with 0.59 μL/s flow rate, excluding cell debris and using the red laser on 1 × 10^4^ events. Data from the untreated cells were used as a reference. Results were analyzed with Guava Incyte Software (Merck Millipore) and displayed in terms of MFI events, characterized by a shift in the Red-R fluorescence intensity.

Experiments were carried out at least twice for each treatment and each cell line.

### 2.6 Statistical Analysis

The statistical analysis between the analyzed groups was performed with the two-way analysis of variance (ANOVA, normality test Shapiro–Wilk, equal variance test Brown–Forsythe) or *t*-test tools of SIGMA Plot software’s data analysis package. **P ≤ 0.001 and *P ≤ 0.05 were considered significant. Results were represented as mean ± SE or mean ± standard deviation (SD).

## 3 Results

### 3.1 Characterization of Extracellular Vesicles

**Figure 1:**
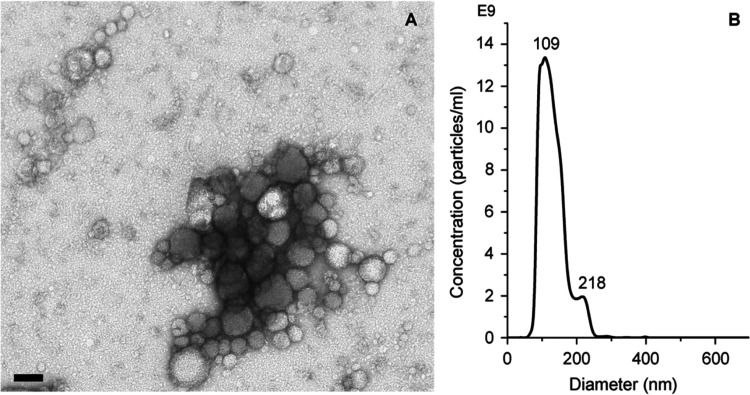
Panel (A) represents a TEM image of EVs and (B) NTA representative size distribution graph. Scale bar: 100 nm.

The TEM analysis of the just isolated EVs revealed the presence of a heterogeneous population of round-shaped vesicles, with a thin and electron-dense membrane and size ranging from 50 to 150 nm (Figure 1A). By NTA, the average concentrations (1 × 10^11^ ± 6 × 10^10^ part/mL) and the average diameter (the mean diameter was 138 ± 6 nm, the mode 108 ± 10 nm) of vesicles from 15 different isolations were calculated (Figure 1B). Bradford assays on the same isolations returned a total protein concentration of 140 ± 40 μg/mL and an EVs purity ratio of 8 × 10^8^ ± 3 × 10^8^ part/μg. EVs’ surface antigen expression was detected as MFI, and the results, in agreement with those reported in literature,^39,45,46^ were respectively 0.5 ± 1.1 for CD63, 19 ± 4 for CD20, and 3.1 ± 1.0 for CD81. Since the best expressed antigen was found to be CD20, the expression of the latter was used to test the effect of the different storage methods on the biological activity of the isolated EVs.

### 3.2 Evaluation of the Effects of the Storage Conditions on the EVs’ Size and Biocomposition

To evaluate the effects of the storage methods on EVs, different methods were combined. In detail, NTA was used to observe an increase or decrease in the EVs sample’s concentration, which indicated damages due to fragmentation or aggregation of EVs. The analyses with Bradford assay highlighted fluctuations in the sample’s protein concentration; an increase meant EVs rupture and leakage of luminal proteins, while a reduction corresponded to possible aggregation of the EVs’ membranes. Then, the influence on the EVs’ membrane composition was investigated through the analysis of the expression of the CD20 antigen, whose variations could indicate membrane damage.

**Figure 2:**
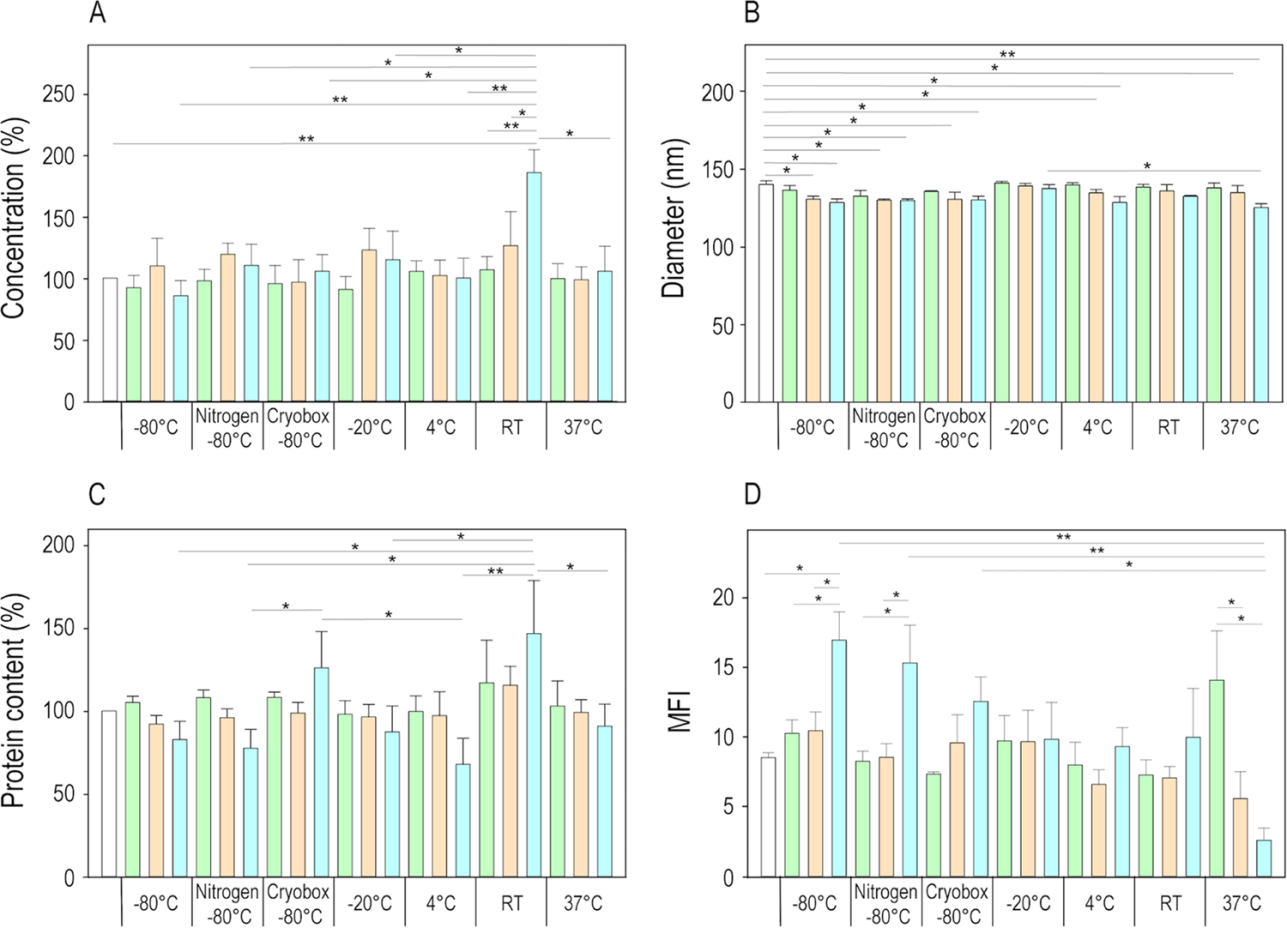
(A) NTA concentration, (B) EVs mean diameters, (C) Bradford analysis, and (D) CD20 antigen expression results of EVs samples stored at different temperatures after 1 day (green bars), 1 week (orange bars), and 1 month (blue bars). NTA and Bradford analysis data were represented as a percentage of the fresh, just isolated sample (white bar). Graphs were plotted as mean ± SE. Independent experiments were carried out in triplicate, and ANOVA test was used for the statistical analysis, * for *P* ≤ 0.05 and ** for *P* ≤ 0.001.

As shown in Figure 2A, NTA measurements showed that after 1 day and 1 week, the EVs samples stored with different methods maintained the particle concentrations like the fresh sample. By contrast, after 30 days, the RT preservation method strongly affected the integrity of EVs, if compared to the fresh sample (*P* ≤ 0.001) and samples stored at −80 °C (*P* ≤ 0.001) or in nitrogen and then at −80 °C (*P* = 0.005) or in a cryobox at −80 °C (*P* = 0.002), −20 °C (*P* = 0.01), 4 °C (*P* ≤ 0.001), and 37 °C (*P* = 0.002). These results suggested that for short storage time, i.e., 1 day and 1 week, the concentration of EVs remained comparable to the just isolated sample, regardless of the storage temperature employed. On the contrary, after 30 days, the storage at RT was the most detrimental, resulting in an increased number of EVs.

Regarding the effect of conservation on the average size of EVs, Figure 2B shows that considering only the temperature, EVs stored at −20 °C were bigger than those frozen in nitrogen and kept at −80 °C (*P* = 0.026). Concerned with the storage time, there was increasing reduction of the EVs’ mean diameter over time (fresh vs 1 day *P* = 0.025, vs 1 week *P* ≤ 0.001, vs 1 month *P* ≤ 0.001; 1 day vs 1 week *P* = 0.022, vs 1 month *P* ≤ 0.001; 1 week vs 1 month *P* = 0.020), a symptom of a general fragmentation of the EVs during the storage. The different interactions between the time and storage method highlighted that ultralow temperatures caused a decrease of the EVs’ mean diameters compared to the fresh samples for time periods of 1 week and 1 month (for −80 °C, *P* = 0.045 and 0.011; for nitrogen and then −80 °C, *P* = 0.028 for both times; for the cryobox at −80 °C, *P* = 0.044 and 0.038). At higher temperatures, i.e., 4 and 37 °C, 1 month-stored EVs samples had smaller dimensions than those of fresh and 1 day-stored samples (for 4 °C, *P* = 0.004 and 0.019; for 37 °C, *P* ≤ 0.001 and *P* = 0.006). In addition, comparing the dimensions of the 1 month-stored EVs at −20 and 37 °C, it was observed that the first ones well tolerate the storage, while the last ones were significantly smaller (*P* = 0.036), being strongly damaged by the prolonged preservation. By contrast, the average size of EVs preserved at −20 °C and RT did not change over time.

The Bradford assay (Figure 2C) confirmed the NTA results; for all the considered temperatures, 1 day and 1 week storage resulted in similar protein contents to the fresh sample, whereas issues started to come out after 1 month. According to the concentration results, the protein content of EVs stored at RT, after 1 month, significantly increased if compared to samples stored at −80 °C (*P* = 0.011), in nitrogen and then at −80 °C (*P* = 0.005), −20 °C (*P* = 0.022), 4 °C (*P* ≤ 0.001), and 37 °C (*P* = 0.038). The increase in the protein content was also observed for EVs preserved in a cryobox at −80 °C vs EVs at −80 °C after freezing in liquid nitrogen (*P* = 0.045) and at 4 °C (*P* = 0.009). In the case of 30 d storage, the increase in the protein concentration could be the consequence of damages in EVs membranes and hence the release of their fragments, including outer compartment proteins and lumen components.

To verify the morphofunctionality of the vesicles’ membrane, the expression of the CD20 transmembrane protein was evaluated; see Figure 2D. According to the previous characterizations, significant differences with the just isolated samples arose after 1 month of storage. In detail, the CD20 expression increased with the preservation at −80 °C (*P* = 0.031 1 month vs 1 day, *P* = 0.025 1 month vs 1 week and *P* = 0.037–80 vs fresh) and −80 °C after freezing in liquid nitrogen (*P* = 0.021 1 month vs 1 day and *P* = 0.019 1 month vs 1 week), while it strongly decreased at 37 °C (*P* ≤ 0.001 1 day vs 1 month and *P* = 0.003 1 week vs 1 month). The analysis of the CD20 antigen expression gave indications about the entity of the damages that occurred to EVs membranes during storage. As observed for vesicle concentration, dimension, and total protein content, until 1 week, the CD20 expression remained comparable to the fresh sample. In contrast, the antigen expression variations after 1 month were caused by damages to the membranes, probably due to prolonged freezing time, as for −80 °C and −80 °C after freezing in liquid nitrogen, or to degradation of proteins at higher temperatures (37 °C).

### 3.3 Freeze-Drying of Extracellular Vesicles

#### 3.3.1 Thermal Characterization of the Various EVs Formulations

The thermal analysis evidenced that the presence of the physiologic solution, containing NaCl, depressed the *T*_g_′ and *T*_c_ of the solutions via plasticization.^47−51^ PS without the excipient showed a *T*_eu_ of around −21 °C^52,53^ and it interacted with the other excipients, modifying their *T*_g_′ or *T*_eu_. In each formulation with amorphous excipients, the *T*_g_′ increased by increasing the ratio between the excipient and NaCl concentration; thus, the 5% w/v sample displayed a lower *T*_g_′ than the one at 10%.^49,54^ In the crystalline formulations, the *T*_eu_ was independent of the concentration. However, we observed a very low collapse temperature for formulations containing mannitol that does not correspond to any of the melting temperatures of the various polymorphic forms of mannitol. This result could be attributed to the formation of amorphous mannitol and the consequent depression of its glass transition temperature promoted by sodium chloride. This aspect should be further investigated by evaluating the crystallinity of the lyophilized samples, e.g., through X-ray diffraction analysis, but it is out of the scope of this study. The collapse temperature of formulations containing lactose, sucrose, and trehalose benefited from the addition of dextran to the formulations, whereas the addition of PEG or glycine did not improve those temperatures. All the results are listed in Table 3. The *T*_c_ of the sample with the physiologic solution was not detectable since the solid content (0.9% w/w) was not sufficient to form a solid cake.

**Table 3:** *T*_g_′, *T*_eu_, and *T*_c_ of the Formulations Measured with DSC and FDM, Residual Moisture Content Analyzed by Karl Fischer Titrator, and Visual Cake Appearance of the Freeze-Dried Formulations Are Listed^a^

excipient	*T*_g_′ (°C)	*T*_eu_ (°C)	*T*_c_ (°C)	residual moisture (%)	cake appearance
PS		–21	ND	1	good
L5%	–45		–47	10	collapsed
L10%	–37		–29	5	collapsed
S5%	–47		–40	8	collapsed
S10%	–39		–33	7	collapsed
T5%	–47		–40	10	collapsed
T10%	–39		–41	10	collapsed
C5%	–37		–38	3	good
C10%	–27		–24	<1	good
D5%	–27		–24	<1	good
D10%	–19		–15	<1	good
L-D	–33		–29	<1	good
S-D	–36		–34	<1	good
T-D	–34		–31	<1	good
D-PEG	–29		–24	<1	good
L-PEG	–34		–41	3	collapsed
S-PEG	–45		–42	2	collapsed
T-PEG	–43		–40	4	collapsed
M5%		4	–45	<1	good
M10%		3	–38	<1	good
G5%		–25	–39	ND	good
G10%		–25	–24	ND	good
L-G	–47		–47	ND	collapsed
S-G	–48		–44	ND	collapsed
T-G	–50		–45	ND	collapsed
HS10%		–23	–24	<1	good
HS50%		–24	–23	<1	good
HSDC10%		–23	–24	<1	good
HSDC50%		–24	–23	<1	good
RPMI10%		–23	–40	<1	good
RPMI50%		–24	–40	<1	good
Secretome10%		–23	–36	<1	good
Secretome50%		–25	–31	<1	good

a ND indicates non-detectable samples.

#### 3.3.2 Residual Moisture

The lyophilization cycle must be accurately designed to guarantee that the residual water content of the lyophilized cake is within a precise range of values, typically 1–3%, to guarantee the long-term stability of the active pharmaceutical ingredient (API), especially in the case of large-molecule formulations and certainly in the case of complex biostructures such as EVs. Furthermore, the control of the residual product moisture is strictly required since it must ensure an elegant cake appearance and API’s structural integrity while avoiding its degradation processes such as aggregation, deamidation, oxidation, and other pathways that can occur in solution.

**Figure 3:**
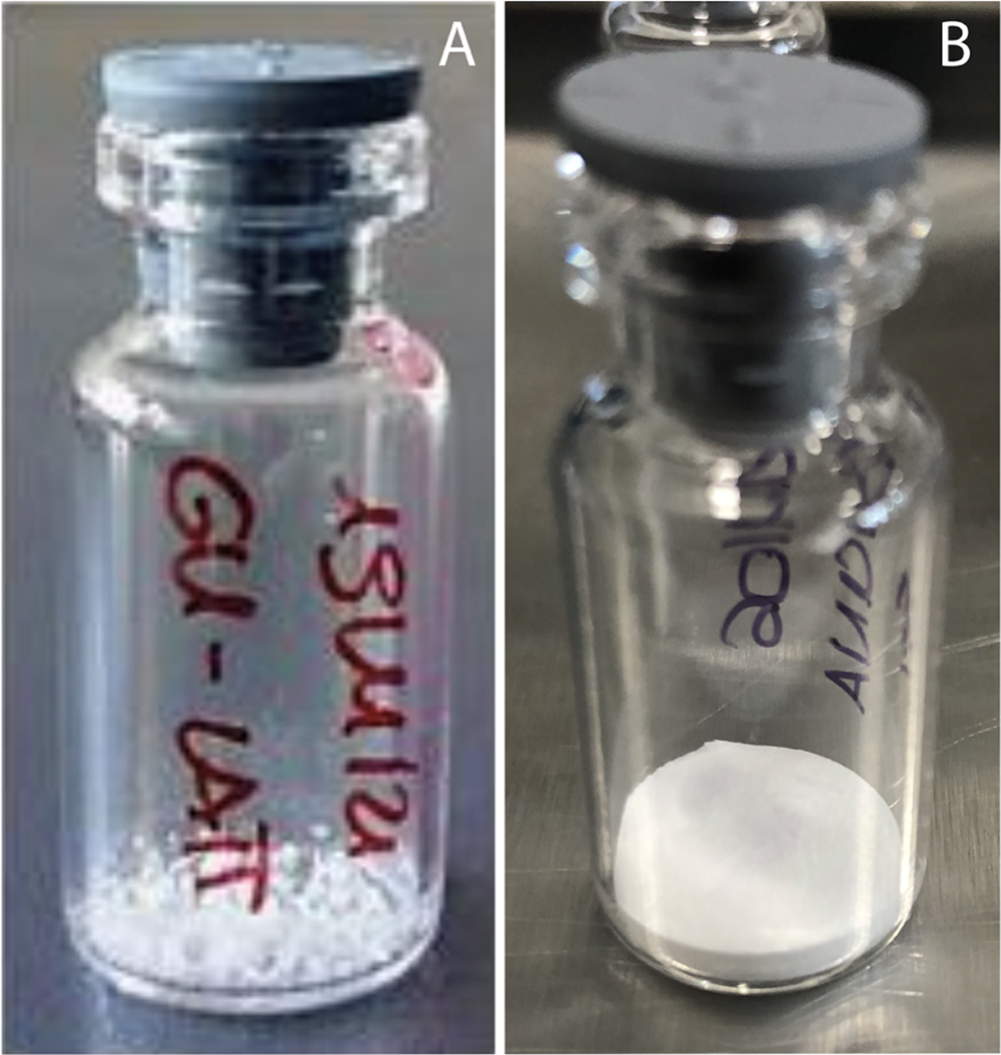
Representative images of (A) collapsed (EVs with lactose and glycine) and (B) good (EVs with glycine 5%) structure of the lyophilized cakes.

An efficient freeze-drying process provides products with a residual moisture (RM) content below 2%, avoiding the action plasticizer, which can lower the *T*_g_ of the lyophilized product.^55^ All the formulations with biological excipients, 10% mannitol, 10% cyclodextrin, and dextran had residual moisture below 2%. In contrast, those formulations containing only sugars or in combination with PEG and 5% cyclodextrin showed an RM above 2%, as reported in Table 3. This result was expected as amorphous materials tend to hold residual moisture back, slowing its release rate. These results had a dramatic impact on the lyophilized cake’s appearance. Formulations with RM >2% corresponded to not formed or collapsed cakes (Figure 3A), while homogeneous and compact structures were observed in the case of lower residual water content (Figure 3B). This result was expected because collapsed samples tend to retain water during the secondary drying phase.

#### 3.3.3 Characterization of the Reconstituted Freeze-Dried Extracellular Vesicles

**Figure 4:**
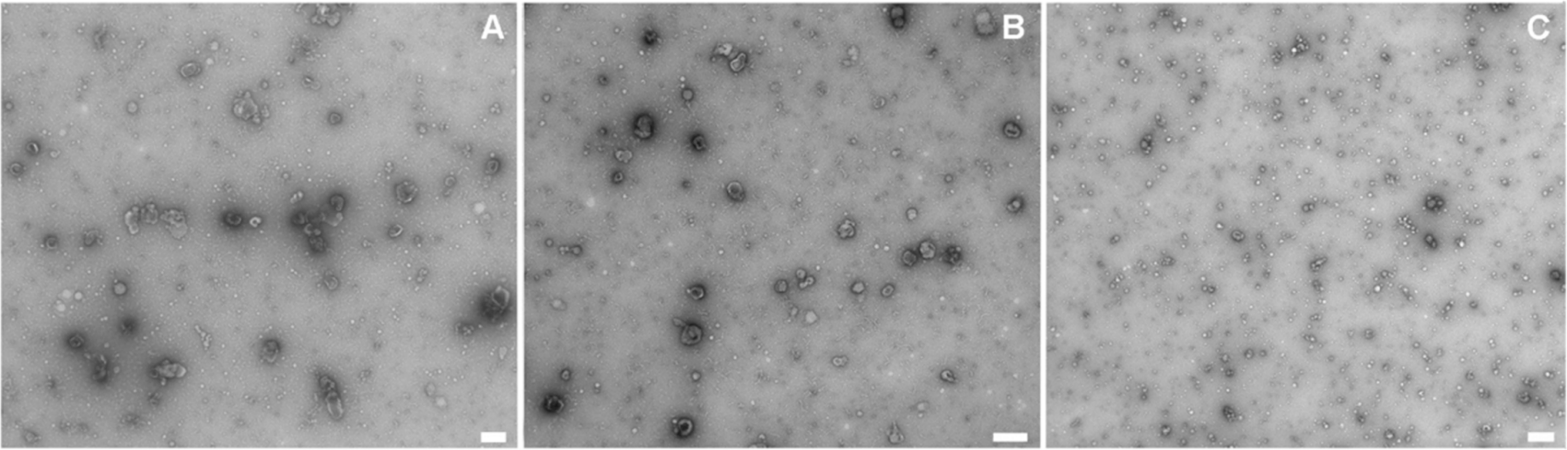
Representative images of EVs (A) just after isolation, (B) after 1 month at −80 °C, and (C) in the case of freeze-drying without excipients. Scale bars are 200 nm.

TEM images were acquired for fresh EVs (Figure 4A), EVs stored at −80 °C for 1 month (Figure 4B), and lyophilized EVs in a physiologic solution (Figure 4C). The freeze-dried samples showed vesicles with a smaller dimension than the fresh and the frozen ones and an increased number of small structures that could be identified as membrane fragments, proteins, or lipid aggregates due to the damage that EVs underwent during freeze-drying if processed in the absence of cryo- and lyoprotectants.

After the reconstitution for the in vitro experiments, EVs were diluted in a physiological solution, and their concentration and size distribution were analyzed with NTA. The results of these analyses are summarized in Table 4. Since the concentration of each isolation batch was different, the concentration of the final reconstituted products was normalized to that of the aliquots stored at −80 °C, making reliable comparisons. In addition, to have a robust statistical analysis, samples with a SE above 30% of the mean value were not considered.

**Table 4:** NTA Results in Terms of EVs Concentration and Average Size Distribution

excipient	concentration (%)	dimension (nm)
–80 °C	100 ± 0	127.2 ± 1.4
PS	47 ± 6	120 ± 4
L5%	60 ± 17	132.8 ± 1.3
L10%	49 ± 13	128 ± 5
S5%	60 ± 20	133.2 ± 1.8
S10%	60 ± 19	126 ± 3
T5%	46 ± 10	130.5 ± 1.4
T10%	70 ± 17	123 ± 3
C5%	62 ± 7	112 ± 5
C10%	62 ± 16	112 ± 3
D5%	33.2 ± 1.0	143 ± 13
D10%	77 ± 7	136 ± 2
L-D	49 ± 14	138 ± 3
S-D	70 ± 20	139 ± 6
T-D	39 ± 4	146 ± 6
D-PEG	91 ± 3	251 ± 81
L-PEG	51.7 ± 0.2	118 ± 20
S-PEG	254 ± 113	124 ± 13
T-PEG	68 ± 18	131 ± 8
M5%	59 ± 15	130.3 ± 1.7
M10%	31 ± 8	126 ± 9
G5%	55 ± 7	133 ± 7
G10%	103 ± 30	126 ± 2
L-G	89 ± 28	126 ± 6
S-G	109 ± 27	136 ± 4
T-G	66 ± 22	134.7 ± 1.6
HS10%	127 ± 8	154 ± 7
HS50%	368 ± 143	147 ± 7
HSDC10%	148 ± 31	155 ± 8
HSDC50%	236 ± 43	151 ± 17
RPMI10%	63 ± 9	147 ± 27
RPMI50%	66 ± 10	163 ± 19
Secretome10%	100 ± 39	125 ± 17
Secretome50%	199 ± 53	101 ± 8

The concentration results were compared with the frozen sample only stored at −80 °C without freeze-drying and with the physiologic solution-lyophilized one without any cryo- or lyoprotectant, using the paired *t*-test. As expected, the PS sample displayed a significantly decreased concentration of EVs if compared with the sample stored at −80 °C (*P* = 0.013); considering that the dimension did not significantly vary, there was probably a loss of EVs during the process due to the absence of cryo- and lyoprotectants. The other formulations, containing 5% (w/v) of excipients, i.e., trehalose (*P* = 0.033), cyclodextrin (*P* = 0.031), dextran (*P* = 0.010), and glycine (*P* = 0.025), and the combinations of trehalose and dextran (*P* = 0.004), and of lactose and PEG (*P* = 0.002) did not successfully protect EVs from the stresses of the freeze-drying process, causing degradation and loss of EVs, with eventual aggregation and fragmentation. In contrast, lyophilized EVs with dextran at 10% (*P* = 0.022), human serum at 10% (*P* = 0.030), and decomplemented human serum at 50% (*P* = 0.048) presented a significantly increased concentration in comparison to the samples with PS, with values more similar to the frozen samples, a sign that these excipients can successfully preserve the EVs.

Concerning the average size of EVs, dextran at 10% and cyclodextrin at 10% displayed different diameters compared to those at −80 °C; in detail, D10% had bigger dimension (*P* = 0.010), while C10% had smaller diameters (*P* = 0.031), a hint of aggregation or fragmentation, respectively, whereas samples lyophilized with lactose at 5% (*P* = 0.031), trehalose at 5% (*P* = 0.049), human serum both at 10% (*P* = 0.034) and 50% (*P* = 0.030), and decomplemented human serum at 10% (*P* = 0.018) displayed bigger diameters compared to PS.

In general, the NTA analyses (NTA control representative images are reported in Figure S1) showed that EVs samples prepared with the addition of biological-derived excipients had a higher compositional and dimensional complexity, due to the presence of nanostructures in many cases close to or below the resolution limit of the investigation technique comprising biomolecules, supramolecular complexes, extracellular matrix components,^56^ and nonvesicular cellular origin nanoparticles such as exomeres and supermeres that can be isolated only with ultracentrifugations ranging from 150 000 to almost 400 000*g*.^57^ In detail, upon lyophilization, EVs with biological-derived excipients had higher concentrations, due to the presence of nanostructures and nanoparticles not discarded from the excipients and dimensions, due to the protein corona formation around the EVs.^58,59^

On the contrary, although dextran and glycine at low concentration (5% w/v) did not properly preserve the EVs initial characteristics, if used at high concentration (10% w/v) or in combination with sugars, they support the integrity of extracellular vesicles during the process.

**Figure 5:**
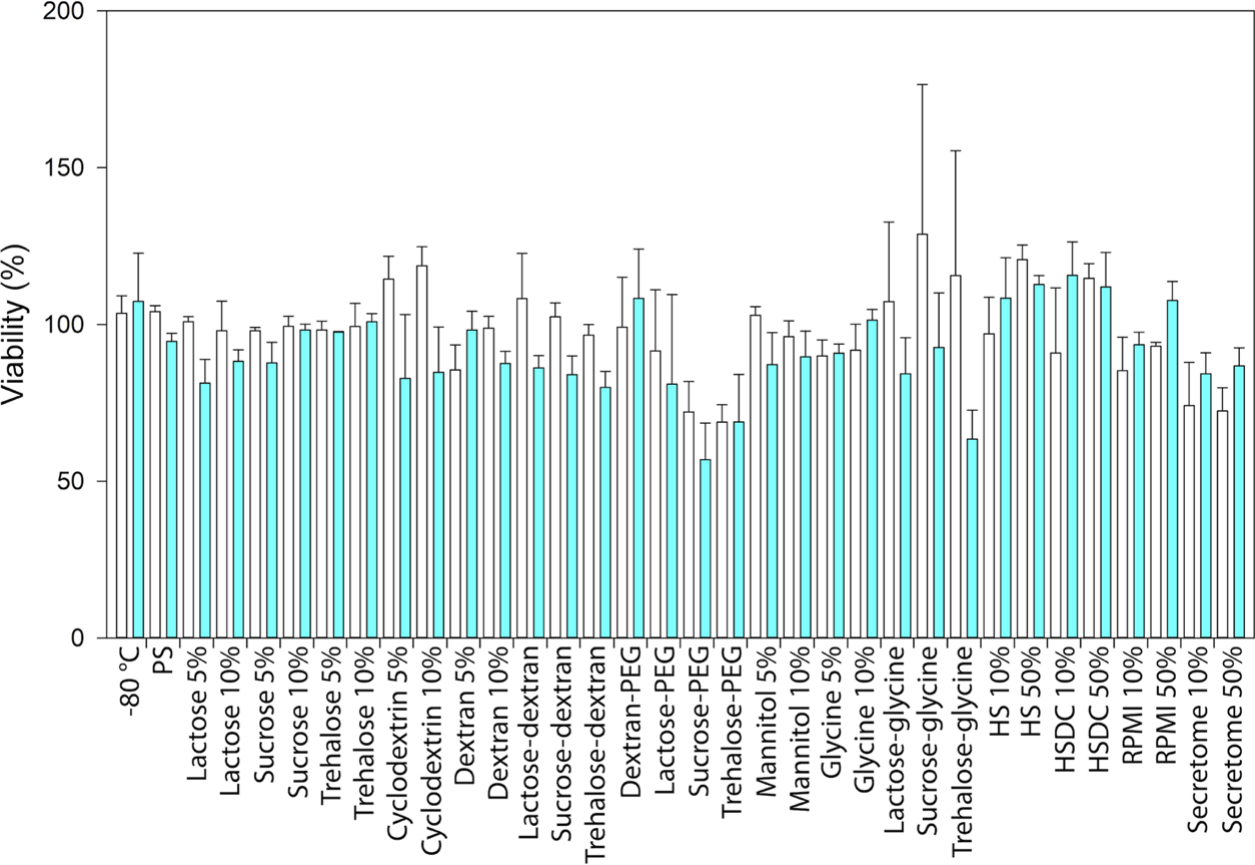
Cytotoxicity assay of freeze-dried EVs after 24 h of incubation in lymphocytes (white bars) and Daudi (blue bars). Independent experiments were performed at least in duplicate.

**Figure 6:**
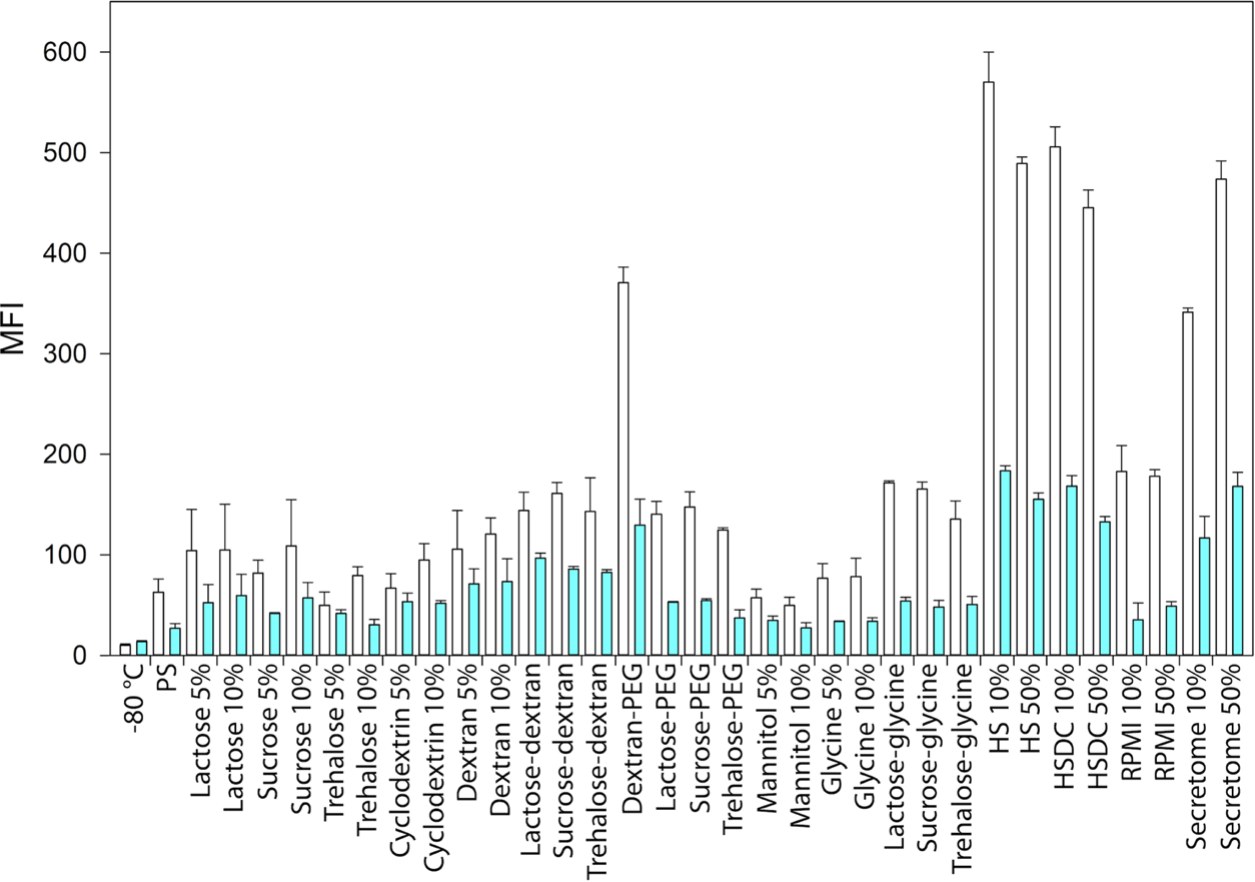
Uptake of freeze-dried EVs after 24 h of incubation in lymphocytes (white bars) and Daudi (blue bars). Independent experiments were performed at least in duplicate.

The biological behavior of the reconstituted lyophilized EVs was evaluated *in vitro* on the parental healthy cell line, B-lymphocytes, and their tumoral lymphoid counterpart, Daudi, in terms of cytotoxicity (Figure 5) and cell internalization (Figure 6).

As shown in Figure 5, the ANOVA test on the cytotoxicity assay pointed out that the freeze-dried EVs impaired more the cancerous cell line, Daudi, compared to the healthy one, lymphocytes (*P* = 0.025).

**Figure 7:**
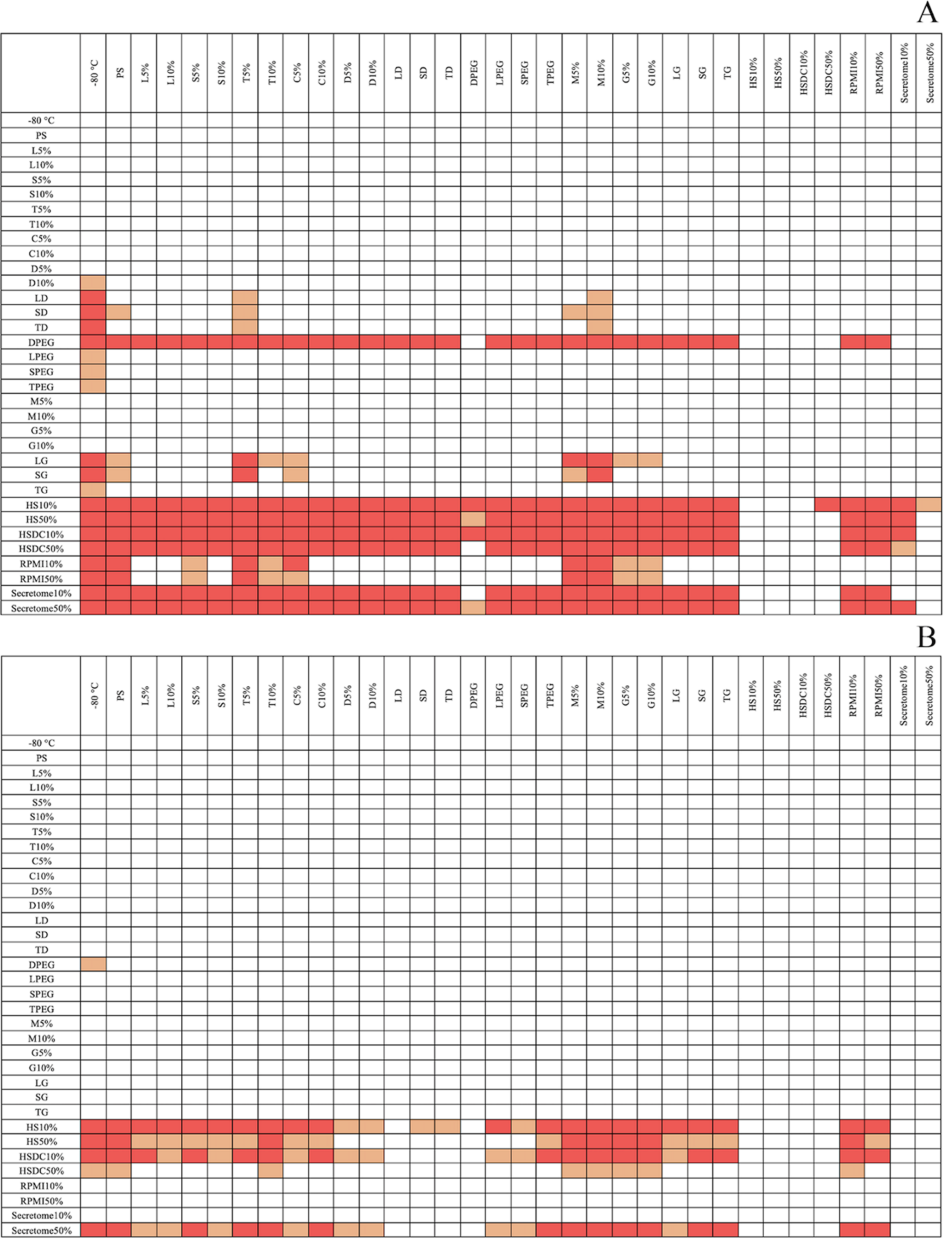
Internalization assay result report of all statistical comparison significances: heat maps representing the statistical analysis of the uptake results using the two-way ANOVA. (A) Lymphocytes. (B) Daudi cells. The red color is for *P* ≤ 0.001, with orange for *P* ≤ 0.05 and white for comparisons with no significant differences.

The results of the *in vitro* cell internalization tests and the related two-way ANOVA statistical analysis are reported in Figures 6 and 7, respectively. As for the cytotoxicity evaluation, a statistically significant difference in uptake resulted considering the cell lines as a source of variation (*P* ≤ 0.001). In detail, considering at the same time the cell line and the different excipients used for their treatments, the lymphocytes’ uptake resulted higher than the one measured for Daudi cancerous cells for 5% lactose (*P* = 0.031), sucrose, trehalose, and 10% dextran (*P* = 0.031, 0.041, and 0.049 respectively), for the combinations of sugars with dextran (*P* = 0.048 with lactose, *P* = 0.002 with sucrose, *P* = 0.012 with trehalose), with PEG (*P* ≤ 0.001 with dextran, *P* = 0.003 with lactose and trehalose, *P* = 0.002 with sucrose) and with glycine (*P* ≤ 0.001) and for the biological-derived excipients (*P* ≤ 0.001).

Considering only lymphocytes, EVs lyophilized with biologically derived excipients, combinations with dextran, glycine, and PEG or dextran at 10%, were significantly more internalized than frozen EVs (*P* = 0.018 for dextran 10%, *P* = 0.005 for L-PEG, *P* = 0.002 for S-PEG and T-G, *P* = 0.040 for T-PEG, *P* ≤ 0.001 for the others). In addition, samples with biologically derived excipients (*P* ≤ 0.001), D-PEG (*P* ≤ 0.001), sucrose–dextran (*P* = 0.020), lactose, and sucrose with glycine (*P* = 0.004 and 0.010, respectively) were uptaken more than their control without excipients but only physiologic solution. Comparing the different excipients, samples containing human serum, decomplemented or not, and secretome at 10 and 50% and dextran–PEG were the EVs samples most internalized by cells, followed by RPMI at 10 and 50%, glycine with sucrose and lactose and the combinations with sugars and dextran; see the heat map in Figure 7A for further information.

Furthermore, considering only the Daudi cells, freeze-dried EVs in the presence of human serum, decomplemented or not, both at 10 and 50%, secretome at 50%, or dextran–PEG were internalized significantly more than not processed EVs (*P* = 0.007 for HSDC50%, *P* = 0.049 for D-PEG, *P* ≤ 0.001 for the others). Among the just listed ones, samples lyophilized with biologically derived excipients were uptaken more than the sample without excipients (*P* = 0.008 for HSDC50%, *P* ≤ 0.001 for the others). The direct comparison among the different excipients pointed out that in general, EVs lyophilized in the presence of HS10% and HS50%, HSDC10%, and Secretome50%, followed by the ones with HSDC50%, were internalized significantly more than most of the other samples, as displayed in Figure 7B.

## 4 Discussion

The morphological structure of EVs is one of the most important issues related to their physiological role and biodistribution.^60,61^ Our study demonstrated that lymphocyte-derived EVs underwent morphofunctional changes during storage since considering different factors such as concentration, average size, protein content, and antigen expression, significant time-dependent differences emerged as reported in the literature.^62^ More in detail, for short storage times, i.e., 1 day or 1 week, EVs maintained their properties at mostly all the preservation temperatures but probably over-zero temperature can be preferred to avoid freezing and thawing cycles.^9^ By contrast, the stability of EVs was compromised in the case of a long storage time, i.e., 1 month, when almost all the preservation temperatures induced some morphofunctional variations. These variations can be due to different mechanisms. The frozen samples can be damaged by the cryoinjury, a combination of osmotic imbalance, which creates a gradient, causing the water to flow out of the vesicles through exosmosis and intravesicular ice formation, resulting in EVs rupture and fragmentation. The first phenomenon is more visible for slow freezing rates, while the second one is for faster freezing, such as nitrogen dipping.^15^ At higher temperatures, proteins and membranes suffer by the action of different degradation pathways. Referring to the proteins’ temperature stability, many authors reported that chemical denaturation at 25 °C is a reversible phenomenon characterized by a Δ*G* decreasing upon increasing protein concentration. Isothermal calorimetry provides data on the effect of how heat variation affects the proteins’ unfolding, aggregation, and precipitation. As for lysozyme also, for many other proteins, denaturation precedes aggregation.^63^ Furthermore, some proteins may also partly display denatured states that can aggregate.^64,65^

The preservation of a biopharmaceutical’s feature during its storage is an essential requirement for the drugs’ clinical use; furthermore, solid forms of drugs are more stable than liquids.^66^ With about 34% of products on the European market, lyophilization is the most employed method to dehydrate biopharmaceuticals, improving their shelf-life, facilitating the handling and transport phases, and reducing the requirements for cold chain maintenance.^67−69^ Nevertheless, the generation of freezing and drying stresses during the process can alter the stability and integrity of EVs,^36^ as also demonstrated by our results. Our work investigated freeze-drying as an alternative approach to cryopreservation; the process removes water from temperature-sensitive products without damage, stabilizing and hence increasing their shelf-life and thus solving the drawbacks of the cold chain interruption. Freeze-drying has a crucial role in the preservation and manufacturing of pharmaceutical products, among which the extracellular vesicles must be considered both as cellular biopharmaceutical derivatives or as nanosized carriers of biocomponents and/or drugs. In a dosage form, even the extracellular vesicles, intended as an API, must be mixed up with excipients to protect them during the various stages of the freeze-drying process without affecting their bioavailability and stability.

The addition of cryo- and lyoprotectants from different origins demonstrated to overcome this issue.^70,71^ Hence, we evaluated the effects of the most used excipients with the ones of some biological or cellular-conditioned fluids. First, our results showed that the addition of disaccharides to EVs, i.e., lactose, sucrose, and trehalose, did not provide cakes suitable for pharmaceutical applications,^44^ even in combination with PEG or glycine. The applied freeze-drying protocol, which gave good results for most of the formulations, was not appropriate in these cases. Indeed, the maximum allowable temperature for these formulations is very low due to the depression of glass transition temperatures promoted by sodium chloride. Consequently, it would be necessary to adopt more cautious heating conditions during the primary drying phase and the transition between primary and secondary drying as well as to prolong the times of the various stages of the process to allow for achieving a sufficiently low moisture. For these formulations, it would be necessary to conduct further studies aimed at assessing whether the collapse of the lyophilized matrix may or may not have an impact on the final characteristics of the EVs. In contrast, better results were obtained with disaccharides in combination with dextran, thanks to its ability to increase the collapse temperature of the product. Furthermore, the preservation of the morphological structure of EVs, dextran, and glycine at high concentration and their combination with sugars maintained the initial concentration and dimensions.

Regarding the biologically derived excipients, NTA has not proved to be the most suitable instrument for the evaluation of the morphological preservation of EVs features upon lyophilization due to the high signal noise/ratio related to the compositional heterogeneity in nanoparticles/nanocomposites/nanocomplexes/biomolecules of the excipients.

Since the biological activity preservation is one of the most important aspects after freeze-drying, reconstituted EVs were tested to assess any eventual cytotoxicity toward healthy (B lymphocytes) and tumoral Daudi cell lines and able to impair more the cancerous cells than the healthy ones, which were also the cell source of EVs. These results showed how the method used to reconstitute freeze-dried products induced a significant increase of lymphocytes’ EVs internalization. Actually, in a previously published paper, we demonstrated that the same EVs from lymphocytes, when used without being freeze-dried, were preferentially internalized in Daudi if compared to lymphocytes.^72^ Experimentally, what has been found can be justified by the fact that the lyophilized vesicles used for the treatments were rehydrated in lymphocytes cell culture medium. Most likely, reconstituting EVs for the in vitro tests with lymphocytes’ culture medium, the establishment of a protein corona around EVs rich in substances attractive to lymphocytes was favored. Many authors have already illustrated how a protein corona is naturally shaped around the surface of EVs^58,59^ in biological fluids, affecting vesicle diameter^73^ as well mobility.^74^ In addition, EVs freeze-dried with the presence of biologically derived fluid demonstrated a significantly higher internalization by both cell lines compared to the other samples. This upregulated uptake in the presence of these excipients benefited not only from the reconstitution medium but also from a strong protein corona formed around the lyophilized and reconstituted EVs by the serum, secretome, and RPMI components.^75^ This corona could protect EVs during freeze-drying, reducing the mechanical and osmotic stresses and making lyophilized EVs more appreciated by cells after the reconstitution. To a lesser extent also, the combination of dextran and PEG improved the internalization of lyophilized EVs. Different factors probably caused this result: the neutral charge of PEG and dextran can conceal the negative charge of EVs, avoiding the repulsive effect with cells and making them more attractive. In addition, PEG and dextran hydrated at high degrees, and when EVs were reconstituted in cell culture medium, they could form a hydration corona around EVs, which can effectively enhance cell internalization.

## 5 Conclusions

Identifying and developing new efficient and well-tolerated treatment strategies are not limited to recognizing enzymes and membrane and nuclear channels and receptors. In addition to the drug design and formulation, together with the release characterization and pharmacokinetic and -dynamic studies, the pharmaceutical industry needs the optimization of storage, stability, and in-use shelf-life parameters to avoid compromising the drug’s efficacy and safety while allowing for fast and inexpensive transport.

Translating EVs from basic and preclinical to clinical research requires both the availability of intact and functionally active therapeutic tools and the implementation of reproducible and reliable medium- and long-term preservation methods.^15^

Our study demonstrated that sugars in combination with dextran and glycine successfully maintained the stability and integrity of EVs upon lyophilization. The vesicles once freeze-dried with the excipients described in this work, after the reconstitution, have shown a selective cytotoxic effect toward cancerous cells, providing valuable elements for better aware and targeted EVs use for clinical applications. In addition, it is of considerable interest in nanomedical and pharmaceutical fields the proof with which we want to conclude that from our results, the cellular uptake of the lyophilized EVs seemed to be driven by the reconstitution media and that biofluid as cell culture medium, human serum, and cell culture secretome can tune in vitro cellular processes such as proliferation and internalization.

In parallel with the rapid development of cell-based therapies developed to treat pathologies not addressed by other small molecules or biological drugs, our preliminary results show how many of the biological cell-free media can be considered increasingly efficient solutions for drug manufacturing and delivery. The introduction of new biocompounds and excipients in the formulation of drugs, repositioning or therapeutic switching, must first evaluate the impact on the safety of the final products. Furthermore, biological cell-free media used as an innovative active compound and/or excipients must be evaluated in order to maximize their efficacy and consistency in the manufacturing of the finished pharmaceuticals.

## Supporting Information Available

NTA analyses of the blanks and EVs with biologically derived excipients (PDF)

## Abbreviations

EVs: extracellular vesicles
RT: room temperature
PEG: poly(ethylene glycol)
FBS: fetal bovine serum
P/S: penicillin/streptomycin
dFBS: depleted fetal bovine serum
Q: l-glutamine
PBS: phosphate-buffered saline
PS: physiological saline solution
TEM: transmission electron microscopy
NTA: nanoparticle tracking analysis
BSA: bovine serum albumin
PE: phycoerythrin
CD63-PE: phycoerythrin-conjugated antibody CD63
APC: allophycocyanin
CD81-APC: allophycocyanin-conjugated antibody CD81
CD20-PE: phycoerythrin-conjugated antibody CD20
MFI: median fluorescence intensity
SE: standard error
L5%: lactose in physiologic solution at 5% (w/v)
L10%: lactose in physiologic solution at 10% (w/v)
S5%: sucrose in physiologic solution at 5% (w/v)
S10%: sucrose in physiologic solution at 10% (w/v)
T5%: trehalose in physiologic solution at 5% (w/v)
T10%: trehalose in physiologic solution at 10% (w/v)
C5%: h-β-cyclodextrin in physiologic solution at 5% (w/v)
C10%: h-β-cyclodextrin in physiologic solution at 10% (w/v)
D5%: dextran in physiologic solution at 5% (w/v)
D10%: dextran in physiologic solution at 10% (w/v)
L-D: lactose and dextran in physiologic solution at 7 and 3% (w/v) respectively
S-D: sucrose and dextran in physiologic solution at 7 and 3% (w/v) respectively
T-D: trehalose and dextran in physiologic solution at 7 and 3% (w/v) respectively
D-PEG: dextran and PEG in physiologic solution at 7 and 3% (w/v) respectively
L-PEG: lactose and PEG in physiologic solution at 7 and 3% (w/v) respectively
S-PEG: sucrose and PEG in physiologic solution at 7 and 3% (w/v) respectively
T-PEG: trehalose and PEG in physiologic solution at 7 and 3% (w/v) respectively
M5%: mannitol in physiologic solution at 5% (w/v)
M10%: mannitol in physiologic solution at 10% (w/v)
G5%: glycine in physiologic solution at 5% (w/v)
G10%: glycine in physiologic solution at 10% (w/v)
L-G: lactose and glycine in physiologic solution at 7 and 3% (w/v) respectively
S-G: sucrose and glycine in physiologic solution at 7 and 3% (w/v) respectively
T-G: trehalose and glycine in physiologic solution at 7 and 3% (w/v) respectively
HS10%: cells and EVs-free human serum at 10% in physiologic solution
HS50%: cells and EVs-free human serum at 50% in physiologic solution
HSDC10%: cells, EVs-free and decomplemented human serum at 10% in physiologic solution
HSDC50%: cells, EVs-free and decomplemented human serum at 50% in physiologic solution
RPMI10%: advanced RPMI 1640 at 10% in physiologic solution
RPMI50%: advanced RPMI 1640 at 50% in physiologic solution
Secretome10%: 100k fraction of secretome, cells and EVs-free, at 10% in physiologic solution
Secretome50%: 100k fraction of secretome, cells and EVs-free, at 50% in physiologic solution
DSC: differential scanning calorimetry
FDM: freeze-drying microscopy
*T*_g_′: glass transition temperature of the frozen sample
*T*_eu_: eutectic melting temperature
*T*_c_: collapse temperature
WGA647: wheat germ agglutinin Alexa Fluor 647 Conjugate
ANOVA: analysis of variance
API: active pharmaceutical ingredient
SD: standard deviation
RM: residual moisture
