# Supplementary material for: Comparative Studies of Different Preservation Methods and Relative Freeze-Drying Formulations for Extracellular Vesicle Pharmaceutical Applications
Source: ACS Biomater Sci Eng. 2023 Sep 6;9(10):5871–85. doi: 10.1021/acsbiomaterials.3c00678 (PMC10565719; doi:10.1021/acsbiomaterials.3c00678)
Supplement: Supplementary file 1 — ab3c00678_si_001.pdf [file ab3c00678_si_001.pdf]

Comparative studies of different preservation methods and relative freeze-drying formulations for extracellular vesicles pharmaceutical applications

Francesca Susa[1,‡], Tania Limongi[1,‡,], Francesca Borgione[1], Silvia Peiretti[1], Marta Vallino[2], Valentina Cauda[1] and Roberto Pisano[1,*]*

[1] Department of Applied Science and Technology (DISAT), Politecnico di Torino, Corso Duca degli Abruzzi 24, 10129 Turin, Italy;

[2] Consiglio Nazionale delle Ricerche di Torino, Strada delle Cacce 73, 10129, Turin, Italy.

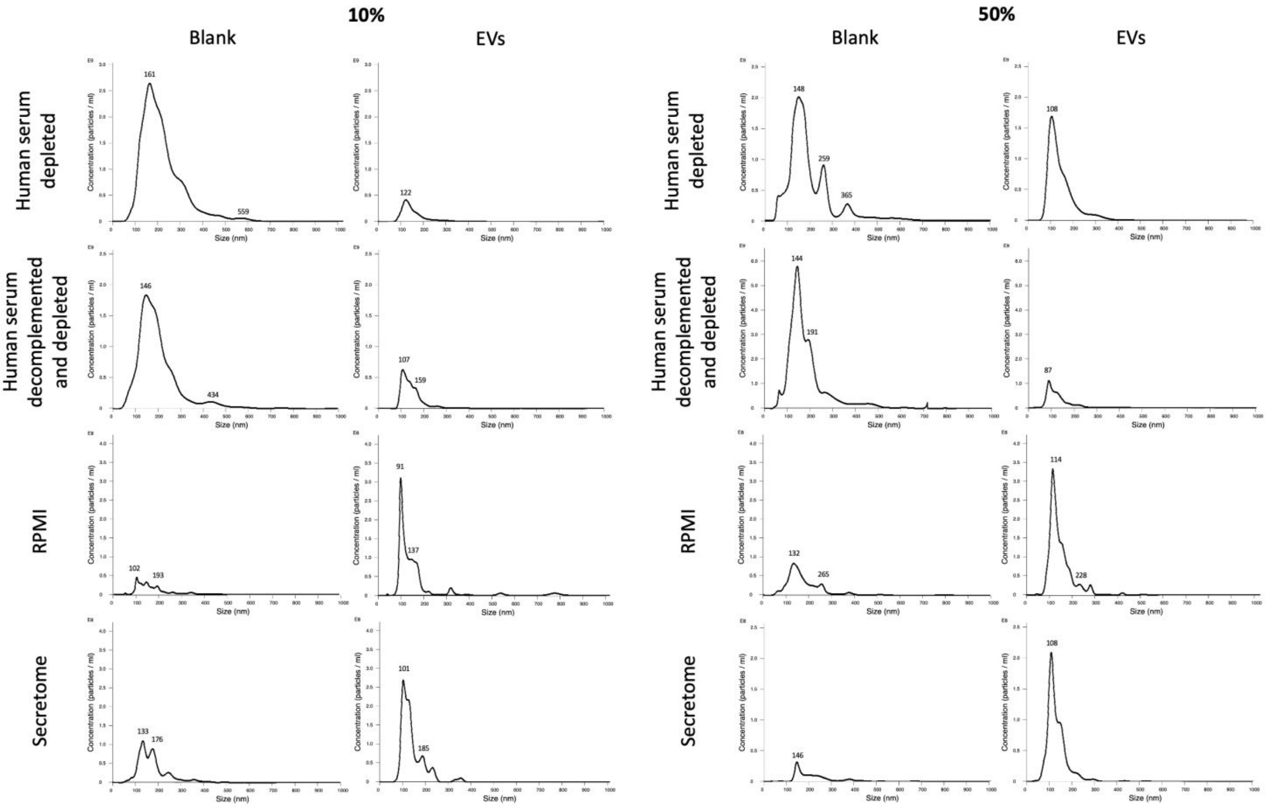

Figure S1. The panel represented the size distribution histograms obtained from the NTA of the samples with biological-derived excipients. In details, the first two columns (blank and sample with EVs) are the samples at the 10% (v/v) concentration, while the latter two (blank and sample with EVs) the ones at 50% (v/v). All the graphs showed the size of the particles (nm) on the x-axis and the concentration (particles/ml) on the y-axis.
